# *Plasmodium berghei*: *Plasmodium* perforin-like protein 5 is required for mosquito midgut invasion in *Anopheles stephensi*

**DOI:** 10.1016/j.exppara.2007.01.015

**Published:** 2007-08

**Authors:** Andrea Ecker, Sofia B. Pinto, Ken W. Baker, Fotis C. Kafatos, Robert E. Sinden

**Affiliations:** Division of Cell and Molecular Biology, Faculty of Natural Sciences, Imperial College London, Sir Alexander Fleming Building, Imperial College Road, London, SW7 2AZ, UK

**Keywords:** Apicomplexa, Malaria, *Plasmodium berghei*, Mosquito, *Anopheles stephensi*, Gene disruption, Ookinete, Mosquito midgut invasion, MACPF, membrane attack perforin, MAOP, membrane attack ookinete protein, MS, mass spectroscopy, MudPIT, multidimensional protein identification technology, *Pb*, *Plasmodium berghei*, PCR, polymerase chain reaction, *Pf*, *Plasmodium falciparum*, PPLP, *Plasmodium* perforin-like protein, PV, parasitophorous vacuole, RT-PCR, reverse transcriptase PCR, SPECT, sporozoite protein essential for cell traversal

## Abstract

During its life cycle the malarial parasite *Plasmodium* forms three invasive stages which have to invade different and specific cells for replication to ensue. Invasion is vital to parasite survival and consequently proteins responsible for invasion are considered to be candidate vaccine/drug targets. *Plasmodium* perforin-like proteins (PPLPs) have been implicated in invasion because they contain a predicted pore-forming domain. Ookinetes express three PPLPs, and one of them (PPLP3) has previously been shown to be essential for mosquito midgut invasion. In this study we show through phenotypic analysis of loss-of-function mutants that PPLP5 is equally essential for mosquito infection. *Δpplp5* ookinetes cannot invade midgut epithelial cells, but subsequent parasite development is rescued if the midgut is bypassed by injection of ookinetes into the hemocoel. The indistinguishable phenotypes of *Δpplp5* and *Δpplp3* ookinetes strongly suggest that these two proteins contribute to a common process.

During the malaria life cycle, three invasive stages are formed, which selectively invade their respective host cells. Invasion is an active process driven by the parasite’s acto-myosin motor and requires secretion from specialised apical organelles, including micronemes and rhoptries (Opitz and Soldati, 2002). In contrast to the merozoite, which invades red blood cells exclusively via the formation of a PV, an alternative mode of cell invasion, termed “cell traversal” or “cell breaching”, is used by the ookinete to cross the mosquito midgut epithelium. Cell traversal does not involve the formation of a PV, probably reflected by the absence of rhoptries in the ookinete (Sinden, 2004), and ultimately results in death of the invaded midgut cell by apoptosis (Han et al., 2000). Sporozoite invasion of hepatocytes occurs first by cell traversal, while entry into the final host cell, in which further development takes place, involves the formation of a PV (Mota et al., 2001).

A protein family implicated in cell traversal are the *Plasmodium* perforin-like proteins (PPLPs), a family of five putative secreted proteins conserved across the *Plasmodium* species (Kaiser et al., 2004). PPLPs are characterised by a MACPF-like domain, which in other proteins has been shown to play a role in the formation of transmembrane channels in lipid bilayers. While direct biochemical proof of a pore-forming activity of the PPLP MACPF domain is still lacking, it has been suggested that this pore formation may either weaken the target cell membrane or allow injection of micronemal proteins into the target cell (Ishino et al., 2005). Accordingly, during the malaria life cycle PPLPs have been detected by MS mainly in the invasive stages (Florens et al., 2002; Lasonder et al., 2002; Hall et al., 2005). As further evidence for a role in cell invasion, at least two family members, PPLP1 and PPLP3, have been shown to localise to the micronemes (Kaiser et al., 2004; Kadota et al., 2004) and the *pplp1*/*spect2* and *pplp3*/*maop* gene disruptions abolished cell traversal in the sporozoite and ookinete, respectively (Kadota et al., 2004; Ishino et al., 2005). However the detection of PPLP2 in *Plasmodium falciparum* merozoites and of PPLP5 in *P. falciparum* gametocytes (Florens et al., 2002) argues for additional roles of PPLPs other than in cell traversal, such as in exit from the host cell.

Ookinete midgut invasion is a major population bottleneck in the malaria life cycle and proteins essential for invasion, such as PPLP3, may be prime targets for transmission blocking vaccines. Besides PPLP3, *P. berghei* ookinetes reportedly express PPLP4 (Hall et al., 2005; Raibaud et al., 2006), and we report here, for the first time, evidence for expression of PPLP5 in the ookinete. PPLP5 was detected by MudPIT in a surface enriched ookinete proteome (R.R. Stanway, unpublished data) and expression was confirmed by RT-PCR (Fig. 1) on cDNA prepared from *P. berghei* gametocytes and purified ookinetes. Interestingly *pplp5* was also amplified from day 5 and day 10 oocyst cDNA, indicating that the gene may be expressed throughout parasite development in the mosquito. This is consistent with data from *P. falciparum*, where PPLP5 was detected by MS in gametocytes and sporozoites (Florens et al., 2002).

In an attempt to understand why the ookinete expresses more than one PPLP protein and to investigate their respective functions, we removed the entire coding region of *pbpplp5* (PB000511.01.0) by double cross-over homologous recombination and integration of a modified *Toxoplasma gondii dihydrofolate reductase/thymidylate synthase* (*dhfr*/*ts*) gene cassette which confers resistance to the antimalarial drug pyrimethamine. Two independent transfections were carried out to generate two independent *Δpplp5* clones, clone 1 and clone 2, which were characterised by diagnostic PCR (Fig. 2a). Successful gene disruption was further confirmed by our failure to amplify *pplp5* mRNA from *Δpplp5* ookinete cDNA (Fig. 2b).

*Δpplp5* parasites showed normal asexual and sexual blood stage development and were able to form ookinetes *in vitro* and *in vivo* in numbers comparable to *wt* (data not shown). However when mosquitoes were allowed to feed on mice infected with *Δpplp5*, no oocysts were observed in midguts dissected on day 10 of infection (Table 1A). *Δpplp5* parasites also failed to infect when ookinetes were cultured *in vitro* and fed to mosquitoes via membrane feeding (Table 1B). Accordingly, no *Δpplp5* sporozoites were observed in salivary glands of these mosquitoes on day 21 of infection. However, the block in infection was not absolute, as we observed a single oocyst each in two of 50 dissected mosquitoes in one experiment. Moreover, in another experiment a single sporozoite was observed in salivary gland dissections (under identical conditions more than 500 were observed in the respective *wt* control). Strikingly, these mosquitoes were able to transmit *Δpplp5* parasites to a C57BL/6 mouse, a mouse strain which is highly susceptible to infection by sporozoites (Jaffe et al., 1990). Diagnostic PCR on genomic DNA prepared from the resulting blood stage infection confirmed that these parasites were indeed *Δpplp5*, indicating that while midgut invasion is almost entirely blocked, the parasites seem to be able to complete the rest of their life cycle.

To test whether bypassing the midgut would thus completely rescue the mutant phenotype, *Δpplp5* ookinetes were cultured *in vitro* and either fed to mosquitoes by membrane feeding or injected into the mosquito hemocoel. Since ectopic oocysts can develop virtually anywhere in the mosquito hemocoel (Paskewitz and Shi, 2005), their quantification is unreliable and we therefore determined salivary gland sporozoite numbers on day 20–22 of infection (Table 2). Hemocoel injection completely restored mosquito infectivity of *Δpplp5* ookinetes, indicating that the block in infection is specifically due to the inability of *Δpplp5* ookinetes to cross the midgut epithelium. Ectopic *Δpplp5* oocysts appeared morphologically normal (data not shown) and, importantly, *Δpplp5* sporozoites were able to infect C57BL/6 mice by tail vein injection and by direct bite-back with prepatent periods similar to *wt*. Both *wt* and *Δpplp5* parasites were first detected in Giemsa stained blood smears 4–5 days post-bite/injection, indicating that sporozoites were fully infectious and both hepatocyte infection and liver stage development were not affected.

To determine more precisely at which point during midgut invasion *Δpplp5* ookinetes were blocked, infected mosquito midguts were also analysed by immunofluorescence microscopy (Fig. 3a–c). Twenty-four hours after infection most *wt* ookinetes had already crossed the mosquito midgut epithelium, reached the basal lamina side and begun rounding up (Fig. 3b). Extruding midgut epithelial cells (Fig. 3a) and upregulation of *Anopheles stephensi* Serpin 6 (Fig. 3b)—both markers for midgut invasion (Han et al., 2000; Abraham et al., 2005)—were also observed in *wt* infected guts. In contrast *Δpplp5* ookinetes were found attached in large numbers to the apical side of the midgut (Fig. 3a) where they persisted (in decreasing numbers) until 48 h post-infection (data not shown). No signs of cell invasion were observed in these guts. In confocal cross-sections of these midgut preparations *wt* parasites were detected within and on the basal side of the midgut epithelium, whereas *Δpplp5* ookinetes remained on the apical side (Fig. 3c). These observations were confirmed by the analysis of toluidine-stained semithin sections of midguts that were fixed 24 h post-blood feed (Fig. 3d). Invasion by *wt* ookinetes had induced massive damage to the midgut epithelium, while in contrast, no invasion of the midgut epithelium by *Δpplp5* ookinetes was observed. Interestingly, *Δpplp5* ookinetes were stuck within the microvilli layer but had successfully crossed the peritrophic matrix.

In summary we have shown that *Δpplp5* ookinetes form normally *in vivo*, that they escape from the blood meal and move to the midgut epithelium, but are incapable of entering the midgut epithelial cells potentially due to a loss of cell-traversal activity. Importantly, if the midgut is bypassed by hemocoel injections of *in vitro* cultivated ookinetes, full infectivity to the mosquito is restored and the parasites are able to complete the rest of their life cycle. Thus, while expression of *pplp5* has also been detected in *P. falciparum* sporozoites in microarray (Le Roch et al., 2003) and proteomic studies (Florens et al., 2002), and in *Plasmodium yoelii* sporozoites by RT-PCR (Kaiser et al., 2004), at least in *P. berghei* it is dispensable at this stage.

While rescue of function by removal of a cellular barrier has not been shown for the *Δpplp3*/*maop* parasite and thus nothing can be concluded about the role of PPLP3/MAOP following midgut invasion (Kadota et al., 2004), full infectivity of *Δpplp1*/*spect2* parasites was restored by Kupffer cell depletion which allowed Δ*plpp1*/*spect2* parasites direct access to hepatocytes (Ishino et al., 2005). Thus, both PPLP1 and PPLP5 play crucial roles only at single and different points in the parasite life cycle. Notably in both *Δpplp1*/*spect2* and *Δpplp5* parasites infectivity was not completely abolished. We observed natural transmission of *Δpplp5* parasites in one experiment, and Ishino et al. report that *Δpplp1*/*spect2* parasites were capable of infecting rats (Ishino et al., 2005). We suspect that this low rate of transmission may occur should the cellular barrier be naturally compromised. Alternatively this low cell-traversal activity may be provided by other members of the PPLP family.

The loss of infectivity of *Δpplp5* ookinetes is striking, considering that the ookinete expresses three members of the PPLP family (PPLP3, 4 and 5) (Hall et al., 2005), and that *Δpplp3*/*maop* ookinetes were equally unable to cross the midgut epithelium (Kadota et al., 2004). The virtually identical phenotype of *Δpplp3*/*maop* and *Δpplp5* ookinetes strongly suggests that these two proteins may interact functionally. Interestingly, the MACPF domain containing late complement components and perforin indeed function as polymers (Peitsch and Tschopp, 1991). If PPLP3 and PPLP5 formed a complex, this would obviously be lost in both individual knockouts. Alternatively, these two proteins may function sequentially in the same pathway. We are currently raising antibodies to test these hypotheses and performing gene disruption experiments to determine the role of the remaining PPLP family members.

## Figures and Tables

**Fig. 1:**
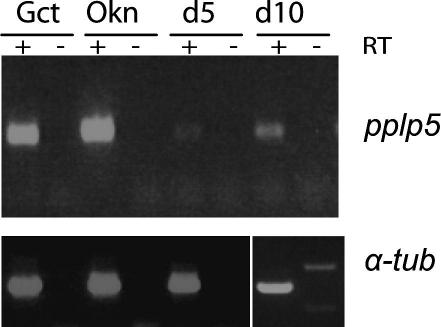
RT-PCR analysis of *pplp5* expression during mosquito development. Routine parasite maintenance in and mosquito infections from Theiler’s Original mice were carried out as previously described (Sinden et al., 2002). *Plasmodium berghei* ANKA 2.34 gametocytes (Gct) were harvested from mice treated for 2 days with sulfadiazine in the drinking water to decrease asexual parasitaemia, and purified by ammonium chloride lysis at 4 °C. Ookinetes (Okn) were cultured *in vitro* and purified using α-Pbs28 antibody (13.1) coupled to magnetic beads (Dynabead) as previously described (Siden-Kiamos et al., 2000; Sinden et al., 2002). Infected *A. stephensi* midguts were dissected on day 5 (d5) or day 10 (d10) of infection. Total RNA was isolated using TRIzol (Invitrogen), contaminant genomic DNA was removed by treatment with TURBO DNA-*free*™ (Ambion) and RNA was cleaned up using the RNeasy Mini Kit (Qiagen). Reverse transcription was performed on 1 μg of RNA using the TaqMan^®^ Reverse Transcription Reagents with a mixture of Oligo-dT primers and Random Hexamers (Applied Biosystems) and the resulting cDNA was used in diagnostic PCRs. Primers N-ter F (5′-TGAATTCATGGGTGATCCACTATTTACT-3′) and N-ter R (5′-TTCTCGAGTTAAAACTTATAACTCTTATATTCATCATC-3′) amplify a 318 bp fragment of *pplp5*, and primers TubF (5′-CCAGATGGTCAAATGCCC-3′) and TubR (5′-CTGTGGTGATGGCCATGAAC-3′) a 432 bp fragment of the α-tubulin gene. + and − denote the presence or absence of RT.

**Fig. 2:**
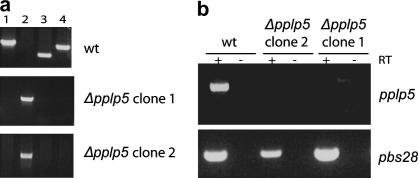
Generation of *Δpplp5* parasites. Generation of constructs for targeted disruption of *pplp5* by double homologous recombination were carried out as previously described (Dessens et al., 1999). Briefly, an upstream homology region of 469 bp was PCR amplified from *Plasmodium berghei* ANKA clone 2.34 genomic DNA using primers AE27A (5′-TT**GGGCCC**GTTGAATATGCATAGACAACATC-3′) and AE27B (5′-CC**AAGCTT**TCACAAATATAGGCTACTCTTGC-3′) and cloned into pBS-DHFR via *Apa*I and *Hin*dIII (restriction sites in bold). A downstream homology region of 570 bp was PCR amplified using primers AE27C (5′-T**GAATTC**TCATATTGAATAGGCCTTATATC-3′) and AE27D (5′-GG**GGATCC**TTTATCACTTCATATCCCAATAC-3′) and cloned into the plasmid with the upstream homology region via *Eco*RI and *Bam*HI. The targeting cassette was released by *Apa*I and *Bam*HI digestion. Parasite transfection using the Human T Cell Nucleofector Kit (amaxa), selection by pyrimethamine and dilution cloning were carried out as previously described (Waters et al., 1997; Janse et al., 2006). Diagnostic PCR (a) on genomic DNA from two independent *Δpplp* clones and control *wt* parasites. PCRs in lane 1 (27KO 5′-TTAGAATATTTTAAGCATTGGCTATC-3′ and 27WT 5′-CAAATGCCAACCAAATGCAC-3′), 3 (N-ter F and N-ter R) and 4 (MACPF-F 5′-TGAATTCGACCCATTTTTTATAAATATGTTGAA-3′ and MACPF-R 5′-TTCTCGAGTTAGCTAGAATAATATTCTAGAGCT-3′) are specific for the *wt* allele. The PCR in lane 2 is specific for integration of the gene targeting cassette (primers 27KO and 248 5′-GATGTGTTATGTGATTAATTCATACAC-3′). RT-PCR analysis (b) of *pplp* expression on total RNA isolated from purified *in vitro* cultivated ookinetes demonstrates absence of transcript in the *Δpplp5* clones. *pplp5* primers as in Fig. 1, p28F (5′-GCGAGATCTATGAATTTTAAATACAGTTTTATTTTTTTA-3′) and p28R (5′-GCGCCTAGCATTACTATCACGTAAATAACAAGTA-3′) amplify the *pbs28* gene (642 bp).

**Fig. 3:**
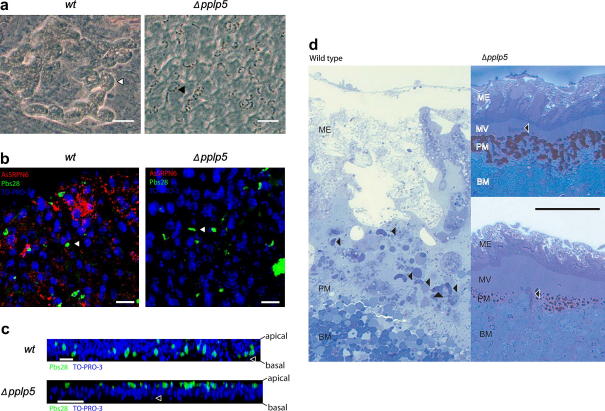
*Plasmodium berghei Δpplp5* ookinetes fail to invade and cross the *Anopheles stephensi* midgut. (a–c) *A. stephensi* midguts were dissected 24 h after feeding of *wt* or *Δpplp5* parasites and epithelia were prepared as previously described (Danielli et al., 2000). Sheets were incubated with purified rabbit α-AgSRPN6 (1:1000) and monoclonal α-Pbs28 (13.1; 1:1000) antibody followed by secondary Alexa-Fluor-488-labelled-goat anti-mouse IgG and secondary Alexa-Fluor-568-labelled-goat anti-rabbit IgG (1:1500, Molecular Probes). Cell nuclei were stained with TO-PRO-3 (Molecular Probes). Samples were analysed using a Leica SP2 confocal (b,c) or a Leica DMR fluorescence microscope and Leica DC500 digital camera (a). Scalebar = 20 μm. (a) Differential interference contrast images show extruding midgut cells (white arrowhead) following invasion by *wt* ookinetes (left), and undamaged gut with large numbers of attached *Δpplp5* ookinetes (black arrowhead) (right). (b) Confocal 3D projection of a *z*-stack shows that *wt* ookinetes have successfully invaded the midgut and started rounding up (green; white arrowhead; left) resulting in upregulation of *A. stephensi* Serpin 6 (red). No Serpin expression was detected in mosquitoes fed with *Δpplp5* parasites (white arrowhead; right). (c) *z*-Stacks show that *wt* ookinetes (green) have crossed the midgut epithelium (top) while *Δpplp5* ookinetes (green) are still found on the apical side (bottom). Open arrowheads indicate nuclei of hemocytes, which are found attached to the basal side of the midgut epithelium. (d) *A. stephensi* midguts were dissected 24 h after feeding of *wt* or *Δpplp5* parasites, fixed as described in Sinden et al. (1985) and semithin sections (500 nm) were prepared and stained with toluidine blue. Images were taken using a Leica DMR fluorescence microscope and Zeiss AxioCam digital camera. Scalebar = 50 μm. Invasion by *wt* ookinetes has caused massive damage to the midgut epithelium (ME) (left), while midguts of mosquitoes fed with *Δpplp5* remain unharmed (right, two examples shown). *wt* ookinetes (arrowhead) are found within the midgut epithelium (left), whereas *Δpplp5* ookinetes (arrowhead) have successfully crossed the peritrophic matrix (PM) but are stuck within the microvilli layer (MV) (right, two examples shown). BM, blood meal.

**Table 1:** Development of *Plasmodium berghei Δpplp5* parasites in *Anopheles stephensi*

	Exp.	Parasite	Oocysts					Salivary gland sporozoites	
			*n*	Prevalence (%)	Mean	SEM	*p*-value	*n*	Mean
A									
	1	wt	52	88	221	22	—	20	4836
		*Δpplp5* clone 1	50	0	0	0	*p* < 0.001	30	0
	2	wt	50	96	111	20	—	30	3104
		*Δpplp5* clone 1	50	0	0	0	*p* < 0.001	30	0
		*Δpplp5* clone 2	50	0	0	0	*p* < 0.001	30	0^a^
	3	wt	50	96	218	31	—	30	5166
		*Δpplp5* clone 1	50	0	0	0	*p* < 0.001	30	0
									
B									
	1	wt	50	100	249	17	—	15	9324
		*Δpplp5* clone 1	25	0	0	0	*p* < 0.001	22	0
		*Δpplp5* clone 2	50	4	0.04	0	*p* < 0.001	13	0
	2	wt	50	98	31	3	—	n.d.	n.d.
		*Δpplp5* clone 1	50	0	0	0	*p* < 0.001	n.d.	n.d.

(A) Direct (gametocyte) feed on infected mice; (B) membrane feeding of *in vitro* cultivated ookinetes; Exp, experiment number; *n*, number of mosquitoes; Prevalence, percentage of mosquitoes with oocysts; Mean, mean number of oocysts or salivary gland sporozoites per mosquito, respectively; SEM, standard error of the mean; *P*-value as determined by *z*-test; n.d., not done; ^a^1 single sporozoite observed.

**Table 2:** Hemocoel injection of *Δpplp5* ookinetes

Parasite	Salivary gland sporozoites					
	Ookinetes fed by membrane feeding			Ookinetes injected into hemocoel		
	Mean	*n*	Infectivity	Mean	*n*	Infectivity
wt	9324^A^	15	Yes^b^	27519	15	Yes^a^
*Δpplp5* clone 1	0^A^	22	No^b^	6670	30	Yes^a^
wt	18492	30	Yes^b^	8794	30	Yes^b^
*Δpplp5* clone 2	0	30	No^b^	14221	29	Yes^b^

Mean, mean number of *Plasmodium berghei* salivary gland sporozoites per *Anopheles stephensi* mosquito; *n*, number of mosquitoes; Infectivity, ability to infect C57BL/6 mice by tail vein injection (a) or mosquito bite (b). ^A^Corresponds to Exp. 1 in Table 1B.
